# Optimized peptide based inhibitors targeting the dihydrofolate reductase pathway in cancer

**DOI:** 10.1038/s41598-018-21435-5

**Published:** 2018-02-16

**Authors:** Amrinder Singh, Neha Deshpande, Nilkamal Pramanik, Siddharth Jhunjhunwala, Annapoorni Rangarajan, Hanudatta S. Atreya

**Affiliations:** 1 NMR Research Centre, Indian Institute of Science, Bangalore, 560012 India; 2 Department of Molecular Reproduction, Development and Genetics, Indian Institute of Science, Bangalore, 560012 India; 3 Centre for BioSystems Science and Engineering, Indian Institute of Science, Bangalore, 560012 India

## Abstract

We report the first peptide based *hDHFR* inhibitors designed on the basis of structural analysis of dihydrofolate reductase (DHFR). A set of peptides were rationally designed and synthesized using solid phase peptide synthesis and characterized using nuclear magnetic resonance and enzyme immunoassays. The best candidate among them, a tetrapeptide, was chosen based on molecular mechanics calculations and evaluated in human lung adenocarcinoma cell line A549. It showed a significant reduction of cell proliferation and an IC_50_ of 82 µM was obtained. The interaction of the peptide with DHFR was supported by isothermal calorimetric experiments revealing a dissociation constant *K*_d_ of 0.7 µM and ΔG of −34 ± 1 kJ mol^−1^. Conjugation with carboxylated polystyrene nanoparticles improved further its growth inhibitory effects. Taken together, this opens up new avenues to design, develop and deliver biocompatible peptide based anti-cancer agents.

## Introduction

In recent years, peptide based therapeutics have emerged as effective alternatives to small chemical entity drugs^1–8^. As a naturally occurring system, peptides possess several favourable characteristics including higher selectivity and potency, which makes them effective even at very low doses^9,10^. This has led to the development of different peptides as anti-cancer drugs^11,12^.

An important target in cancer has been the folate pathway in which the enzyme dihydrofolate reductase (DHFR) enzyme catalyses the reduction of dihydrofolate to tetrahydrofolate using NADPH, which is an essential cofactor for the biosynthesis of purines, thymidylate, and several amino acids^13–15^. Involvement of DHFR in the synthesis of raw material for cell proliferation has made this enzyme an attractive target for various anticancer drugs such as methotrexate (MTX). However, it has been observed that MTX has severe pharmacokinetic problems during the treatment of cancer^16^. Anticancer efficacy of MTX is severely affected by short bloodstream half-life, dose-related side effects, and the development of resistance by cancer cells. The factors that contribute to the resistance includes high intracellular levels of DHFR and loss of the active transport system by which MTX enters cells^17–19^.

In an effort to develop more selective and effective inhibitors of human DHFR (*hDHFR*), we designed and tested small peptide based inhibitors as potential lead compounds. Using a structure-based computational approach based on the crystal structures of *hDHFR* complexed with NADPH and Folic acid [PDB ID: 4M6K], we designed a series of small peptides. We used a similar set of amino acids as those involved in native opioid growth factor (OGF), methionine enkephalin (Tyr-Gly-Gly-Phe-Met), which has been shown to have antitumor activity against a diverse range of cancers^20,21^ and designed various small peptides with slightly different sequences. The synthesis of newly designed peptides was tested for their DHFR inhibition using an *in-vitro* assay and A549 cell-based assays. Efficacy of the designed peptide was further improved upon conjugation with carboxylated polystyrene (PS) nanoparticles (NPs). Nanoparticles are capable of increasing dose of therapeutics inside cells either passively by delivering a larger dose of the drug, or actively through approaches that rely on targeting specific cells^22,23^. In the present study, PS-NPs were chosen as a model system, since they have good cellular compatibility and high stability^24^. Taken together, our results open up new avenues for targeting the folate pathway in cancer.

## Results

### Design of Peptide using molecular docking

**Figure 1:**
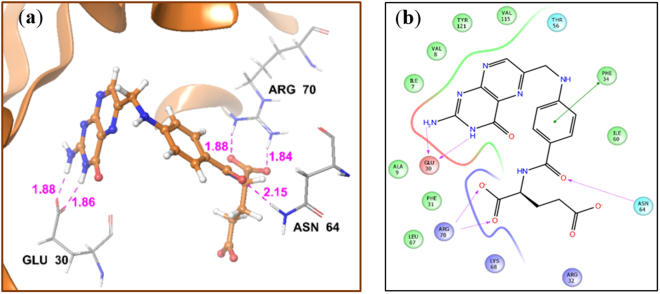
(**a**) Crystal structure of DHFR co-crystallised with Folic acid (orange). Pink dotted lines are H-bonds of folic acid with the various residues, (the distances are given in Å); (**b**) 2D diagram of the binding pose of folic acid in the active site of *hDHFR*.

The peptides were designed by performing molecular docking in the active site of *hDHFR*. Analysis of the crystal coordinates of human DHFR (PDB ID 4M6K) reveals that its substrate (folic acid) exhibits two H-bonds with Glu 30 through the NH_2_ (1.88 Å) and NH (1.86 Å) moiety of pyrimidine ring (Fig. 1); Glu 30 is a key residue for the protonation of substrate and holds the substrate during the catalytic activity of the enzyme^25^. Folic acid also forms two H-bonds with Arg 70 through carboxylate group of glutamate (Fig. 1). In addition, other residues that interact with folic acid inside the active site of the enzyme include Phe 34 (through pi-pi interaction) and Asn 64 (Fig. 1). In order to exhibit potency against human DHFR, the new ligands were designed to maximize contacts with these residues. Before preforming molecular docking of the peptides, the experimentally observed substrate binding mode in *hDHFR* was re-created by docking. For this purpose, the co-crystallized ligand FOL (folic acid) was used as a reference ligand and docked back into its binding site in the crystal structure of *hDHFR* using the GLIDE XP docking program (Schrodinger Inc). The orientation of the ligand obtained after docking closely resembled the co-crystallized conformation with RMSD of 0.9Ᾰ. Following this, the docking protocol was repeated for the peptides.

**Figure 2:**
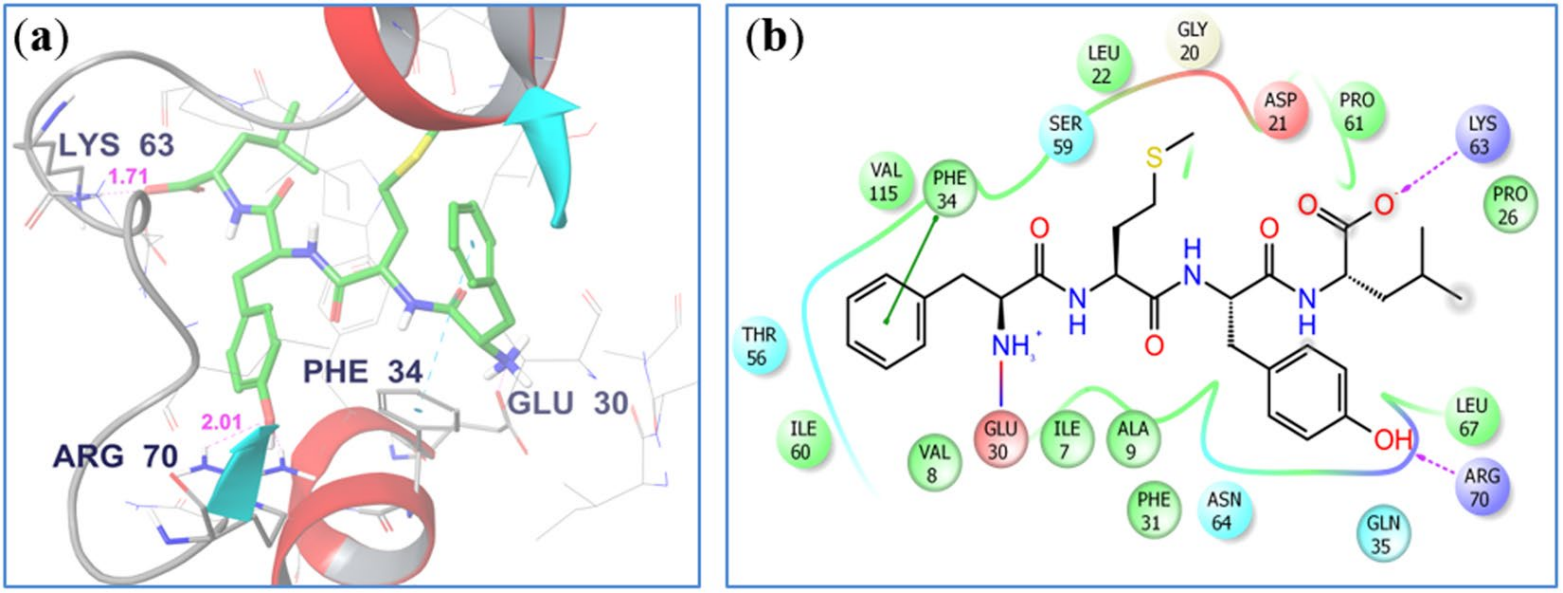
Molecular Docking studies: (**a**) Peptide **2** docked in the active site of *hDHFR*, pink dotted lines are H-bonds. (Non-polar Hs’ are deleted for clarity); (**b**) 2D diagram of the binding pose of peptide **2** in the active site of *hDHFR*.

Table 1 shows the list of peptides designed and corresponding results obtained from docking. When docked in the active site of *hDHFR*, peptide **2** showed a H-bond interaction with Arg 70 (2.01 Å) through the phenolic OH group of tyrosine residue, with Lys 63 (1.71 Å) through carboxyl group of leucine and pi–pi interactions with Phe 34 (Fig. 2). Peptide **2** was able to interact with the same key residues inside active site of *hDHFR* similar to folic acid. However, the binding energy of peptide **2** is the more favourable factor. All other designed peptides were also docked in the active site of *hDHFR* (Table 1). The binding affinities of ligands toward *hDHFR* were determined using Prime/MM-GBSA method based on molecular mechanics calculation (Prime 3.3). The Prime/MM-GBSA calculations were performed using OPLS_2005 force field and VSGB model for polar solvation, leading to the estimation of minimized energies for the protein (G_protein_), the ligand (G_ligand_) and also the protein–ligand complex (G_complex_). The binding free energy of the docked pose was then calculated with: ΔG_bind_ = G_complex_ − G_ligand_ − G_protein_. Being one of the extensively used computational approach, the Prime/MM-GBSA scoring is known to give better correlation with experimental activity data than the docking-based scoring functions^26^.

**Table 1:** Peptides Synthesized for and their IC_50_ values against *hDHFR*.

Peptide no.	Peptide Sequence	% Yield	Theoretical/Experimental mass	Docking score/Binding energy (kcal/mol)	IC_50_ (µM)
1	Tyr-Phe-Met-Leu	78	573.2741 573.2740	−5.4 −28.7	>100
2	Phe-Met-Tyr-Leu	76	573.2741 573.2846	−11.5 −35.6	0.13
3	Phe-Tyr-Met-Gly	77	517.2015 517.1975	−4.9 −27.5	5.6
4	Met-Phe-Tyr-Gly	74	517.2015 517.2012	−3.8 −23.9	>90
5	Tyr-Met-Ser-Leu	68	513.2377 513.2314	−7.2 −28.6	>100
6	Met-Tyr-Ser-Leu	66	513.2377 513.2342	−7.1 −26.5	8
7	Tyr-Phe-Met-Leu-Gly	62	630.2995 630.2831	−7.5 −21.5	>100
8	Phe-Met-Tyr-Leu-Gly	65	630.2995 630.3052	−8.1 −20.7	>100
9	Phe-Tyr-Met-Gly-Leu	69	630.2995 630.3023	−2.7 −15.9	>80
10	Met-Phe-Tyr-Gly-Leu	63	630.2995 630.3029	−1.8 −14.5	>100
11	Tyr-Ser-Phe-Met-Leu	62	660.3031 660.2962	−9.7 −29.9	0.08
12	Tyr-Met-Phe-Ser-Leu	66	660.3031 660.2989	−5.1 −23.7	>90
MTX	Methotrexate	—	—	−12.3 −31.8	0.08

### ITC studies

**Figure 3:**
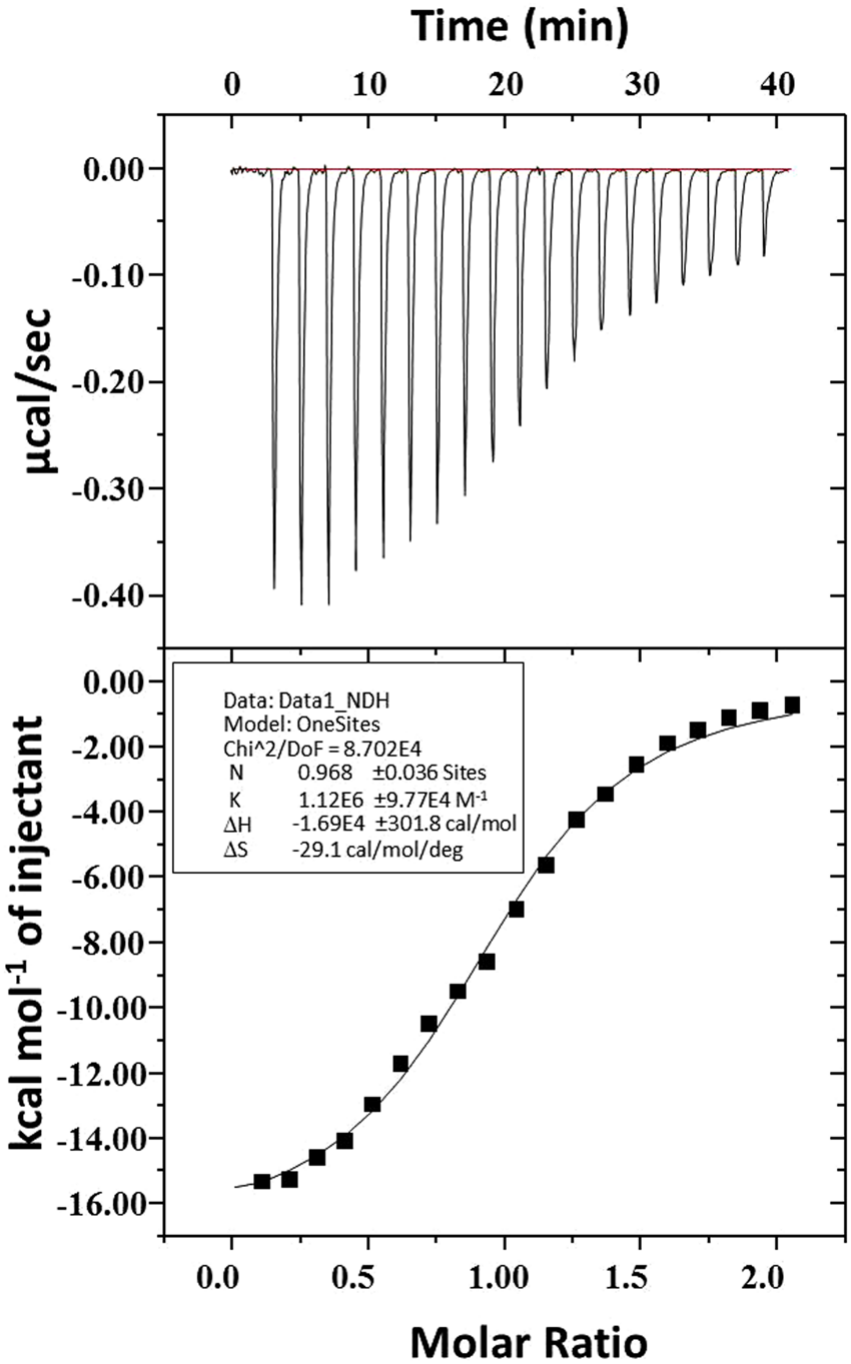
Isothermal calorimetric data of peptide **2** for *hDHFR*.

The binding of peptide **2** with *hDHFR* was further determined using isothermal titration calorimetry (ITC). ITC is considered as one of the most precise techniques to measure the affinity between a macromolecule and a small molecule. Since it measures the heat absorbed or released during complexation, it allows simultaneous determination of all binding parameters with respect to the binding constant (K), enthalpy (ΔH), and entropy (ΔS) in a single experiment. The solution of peptide **2** (in the syringe) was injected into the DHFR enzyme solution taken in the sample cell. In one experiment, 20 consecutive injections of 2 μL of 250 μM peptide were given to the sample cell. Upon each titration, the amount of heat released or absorbed was measured and used to determine the association constant (K_a_), binding enthalpy (ΔH), and entropy (ΔS). The various parameters determined from the ITC experiment of peptide **2** against *DHFR* are given in Table 2 and Fig. 3.

**Table 2:** Isothermal Calorimetric Data of interaction of Peptide **2** with *hDHFR*.

Physical parameters obtained from ITC data	
K_a_(M^−1^)	1.1 ± 0.1 × 10^6^
ΔH (kJ/mol)	−70 ± 1
TΔS (kJ/mol)	−36.25 ± 0.01
ΔG (kJ/mol)	−34 ± 1

### *In Vitro* DHFR Inhibitory Activity

The activity of the designed peptides against *hDHFR* was evaluated by measuring the conversion of dihydrofolic acid to tetrahydrofolic acid in the presence of the test peptide using enzyme immunoassay. Peptides were tested at six different concentrations ranging from 10^−4^ to 10^−9^ M. For comparison, all the peptides under present investigation were screened for *hDHFR* inhibitory activity irrespective of the unfavourable docking results. Supporting the results of the molecular docking studies, peptide **2** and **11** yielded the best IC_50_ values of 0.13 µM and 0.08 µM, respectively, rendering them the most effective inhibitors of *hDHFR* (Table 1). The relatively poor docking score of other peptides was also reflected in their respective DHFR inhibitory activity. Following the results of enzyme immunoassays, peptides **2** and **11** were further evaluated in order to check their potential to inhibit the growth of tumour cells.

### Treatment with peptide **2** inhibits the growth of A549 cells

**Figure 4:**
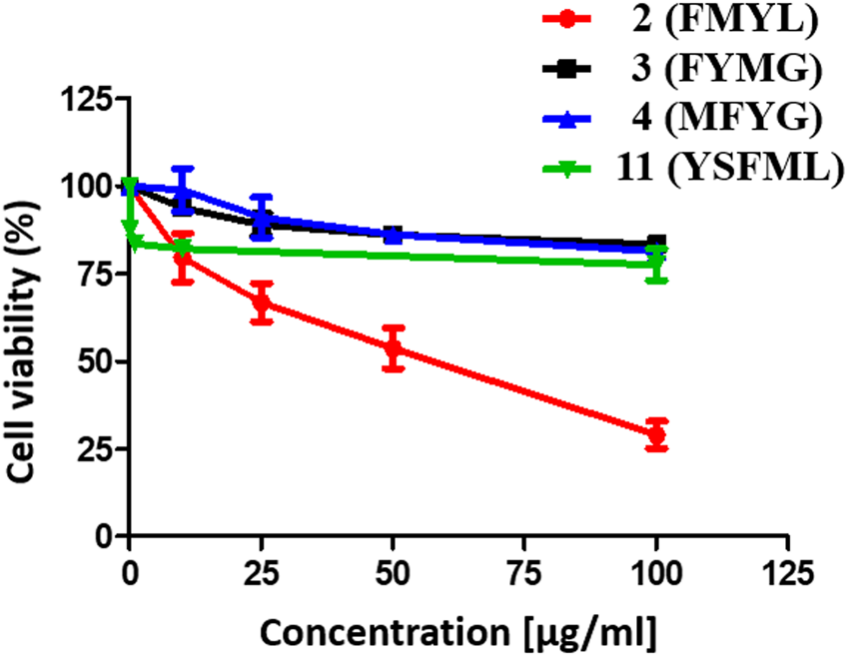
Cytotoxicities of peptides towards A-549 cells: The line graph shows cell viability (%) of A549 cells treated with the indicated concentrations of the various peptides for 48hrs, as determined by MTT assay. Error bars represent mean ± SEM; n = 3.

To validate the efficacy of peptide **2 and 11** *in vitro*, we treated A549, lung adenocarcinoma cells, with increasing concentrations of peptide **2 and 11** for a period of 48 hrs, following which MTT assay was undertaken to determine cell viability. We observed a dose dependent decrease in the cell viability of A549 following treatment with peptide **2** and the IC_50_ of peptide **2** in A549 cells was calculated to be 47 µg/ml (82 µM) (Fig. 4). In spite of having a lower IC_50_ in enzyme immunoassays, peptide **11** failed to show growth inhibitory effects in A549 (Fig. 4). We used yet another approach to corroborate the results obtained with peptide **2**. We monitored the cell confluence using Incucyte ZOOM live cell imaging system for a period of 44 hrs following treatment with increasing concentrations of peptide **2**. Upon treatment with peptide **2**, cell confluence decreased over time in a dose dependent manner as compared to untreated control cells (Supplementary Fig. S8, supplementary video 1 and 2). To eliminate ambiguous interpretation because of non-specific effects of the peptide, we confirmed our results using two other independent peptides having one amino acid difference from the sequence of peptide **2**. Treatment of A549 cells with increasing concentrations of control peptides FYMG (**3)** and MFYG (**4**) did not affect their cell viability (Fig. 4).

### Enhanced growth inhibition of A549 cells by delivery of Peptide **2** via conjugation with polystyrene nanoparticles

**Figure 5:**
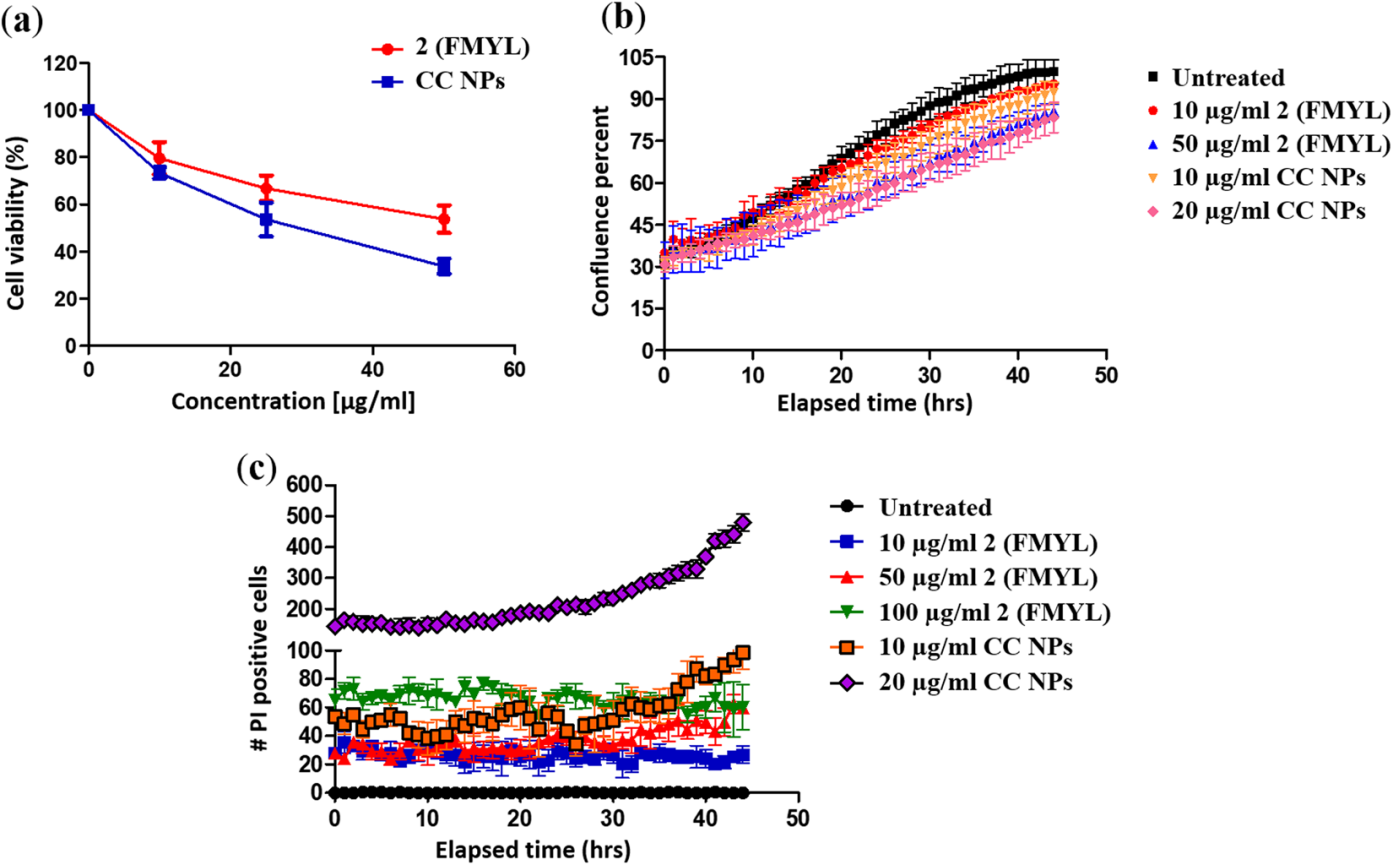
(**a**) The line graph shows cell viability (%) of A549 cells treated, for 48 hrs, with the indicated concentrations of either nascent peptide or peptide conjugated nanoparticles (CC NPs), as determined by MTT assay. Error bars represent mean ± SEM, n = 3. (**b**) Cell confluence percentage of A549 cells was monitored over time following treatment with indicated concentrations of nascent peptide or CC NPs, using Incucyte ZOOM imaging system; n = 3. (**c**) A549 cells treated with differing concentrations of peptide or CC NPs and co-incubated with 1 µg/ml of propidium iodide (PI) were scanned every 1 hr for a period of 44hrs using Incucyte ZOOM imaging system. Graph represents no. of PI positive cells over time. Error bar represents mean ± SEM, n = 3. See also Supplementary Fig. S8, Supplementary videos 1 and 2.

Next, we aimed to enhance the inhibitory effects of peptide **2** on cell viability. To achieve this, peptide **2** was conjugated to carboxylated polystyrene nanoparticles. First, we verified that empty nanoparticles do not affect cell viability (Supplementary Fig. S9). We observed that lower concentration of peptide **2**-conjugated nanoparticles (CC NPs) was required to cause reduction in cell viability as compared to nascent peptide **2** (Fig. 5a). A 20 µg/ml of peptide **2** conjugated with nanoparticles brought about similar decrease in cell confluence as 50 µg/ml of nascent peptide **2** (Fig. 5b). Peptide **2**- conjugated nanoparticles (CC NPs) had an IC_50_ of 34 µM as opposed to 82 µM of the nascent peptide alone. Notably, the number of propidium iodide (PI) positive cells increased upon treatment with nanoparticle conjugated peptide (Fig. 5c). These results identify an effective peptide DHFR inhibitor candidate to be used for chemotherapeutic intervention.

## Discussion

We began our studies by designing small peptides against *hDHFR* using molecular docking. Specifically, the molecular modelling studies helped us in: (i) the design of such peptides that can occupy similar binding pocket in the *hDHFR* active site, which otherwise is occupied by folic acid/MTX and (ii) incorporation of suitable aromatic amino acids to counter the hydrophobic nature of the *hDHFR* active site. The conformational flexibility of the peptides to enter the *hDHFR* active site may help to selectively target the cancer cells due to the higher concentration of the enzyme (i.e., higher expression of DHFR) than in the normal tissues, and advantageously, the side effects associated with the use of *hDHFR* inhibitors as chemotherapeutics may be reduced. The most widely used *hDHFR* anti-cancer agent is methotrexate (MTX). However, MTX suffers from various drawbacks such as development of resistance leading to loss of the active transport system by which MTX enters cells^17^. To improve the properties of MTX, Pignatello *et al*. have reported that conjugation of MTX with shorter-chain α-alkylamino acids increases the lipophilicity of the drug, thus aiding its passive internalization into tumour cells^18^.

Owing to the various problems associated with chemical entity based drugs, the current focus has been on moving toward peptides as therapeutics and approximately 140 peptide based therapeutics are being evaluated in clinical trials^11,12^. Peptide based anti-cancer agents have the potential of providing a means for modulating anticancer activity without affecting the mammalian cells even at relatively higher concentrations. They strongly interact with target proteins with a large surface area; therefore, peptide inhibitors of *hDHFR* may also be effective against MTX-resistant *hDHFR*^27^. *In-vitro* DHFR inhibition assay identified peptides **2** and **11** with an IC_50_ value of 0.13 µM and 0.08 µM comparable with methotrexate whose IC_50_ value, which varies from µM to nM range^28,29^. These two peptides were further evaluated for anti-cancer activity using cell based assays. In the cell based assays using A549 cell lines, the activity was reduced as compared to the *in-vitro* assay probably due to less number of peptide molecules entering into the cells. However, peptide **2** displayed better activity than **11** during cell based assay with an IC_50_ value of 82 µM. The interaction of peptide **2** with *hDHFR* was supported by the results of isothermal calorimetry experiment with a dissociation constant, *K*_d_ of 0.7 µM and ΔG of −34.9 kJ mol^−1^.

Nascent peptides are prone to hydrolysis and oxidation, have a higher tendency to aggregate and undergo faster elimination. Additionally, direct internalization in cells might be low. In order to overcome these problems peptide **2** was conjugated with PS nanoparticles. PS NPs were chosen due to its low toxicity and ease of functionalization with small molecules^30^. This strategy of conjugating peptide **2** with PS NPs resulted in enhanced growth inhibition of A549 cells at lower concentration when compared to peptide in its nascent form and an IC_50_ value of 35 µM obtained.

The optimized and rationally designed peptides thus represent promising novel candidates for targeting the dihydrofolate reductase pathway in cancer. Containing only naturally occurring amino acids, they can be synthesized in pure form using the conventional approach for peptide synthesis. Their delivery to cancer cells and efficacy can be significantly enhanced using biocompatible nanoparticles. This approach can be extended to *in vivo* studies and has the potential to be developed as a lead compound. Taken together, this study opens up new avenues for rational design and delivery of peptide based drugs in cancer.

## Materials and Methods

### Synthesis of peptides: General SPPS procedure

All peptides were synthesized with an automatic solid-phase peptide synthesizer (AAptec) using Fmoc/tBu chemistry. A preloaded 4-benzyloxybenzyl alcohol resin (Wang resin) was used as a solid support for the synthesis of all peptides. First, the Fmoc protecting group on the resin was removed by treatment with 20% piperidine/DMF. The Fmoc-amino acids (3-fold excess) were activated by mixing with the coupling reagent HoBt (3-fold excess), and the resin mixed at room temperature for 10 min. Cycles of deprotection of Fmoc and coupling with the subsequent amino acids were repeated until the desired peptide-bound resin sequence was completed. At the end of the synthesis, the peptide resin was washed with ethanol and dried in vacuo. The protected peptide−resin was treated with a cocktail solution (trifluoroacetic acid/H_2_O/phenol/ethane dithiol 88:5:5:2; v/v; 1 mL/0.1 g of resin) for 1.0 h at room temperature. After filtration of the resin, the solvent was concentrated in vacuo, and the residue was triturated with ether. Crude peptides were purified by preparative RP-HPLC using a Water Delta Prep 3000 system with a Jupiter column C18. The column was perfused at a flow rate of 5 mL/min with a mobile phase containing solvent A (5%, v/v, acetonitrile in 0.1% TFA) and a linear gradient from 0 to 50% of solvent B (80%, v/v, acetonitrile in 0.1% TFA) over 25 min for the elution of peptides. All of the purified peptides were obtained as white powder after lyophilization.

### Formulation of peptide conjugated polystyrene [Pep-g-PS]

Peptide was covalently conjugated on the surface of polystyrene (PS) nanoparticles (200 nm diameter, Bangs Laboratories, USA). In brief, 200 µg of carboxylated polystyrene nanoparticles was treated with 20 mg of EDAC in 1 mL of 0.1 M MES buffer, pH 5.3, for 1 hour. Following this step, 12.1 mg of the NHS was added to the mixture and kept for another 1 hour under constant stirring. Unreacted EDAC and NHS were washed out in the presence of phosphate buffer saline (PBS) solution via centrifugal filtration process. In order to conjugate the peptide, 1 mg of peptide was added to activated-polystyrene particles suspended in phosphate buffered saline (pH7.4) and constantly stirred for 8 hours at room temperature. Finally, conjugated particles [Pep-g-PS] were washed with PBS and stored at 4 °C. The amount of peptide conjugated onto the surface of nanoparticles was calculated by determining the concentration of unreacted peptide molecules at 226 nm and was found to be 62 ± 4 µg (per 200 μg PS particles). Morphology of non-modified particles and peptide conjugated particles was studied using atomic force microscopy (supplementary Fig. S6).

### Molecular docking procedure

The molecular docking was performed using Schrodinger (**Schrodinger Release 2017-1**: Maestro version 11.1). Crystal co-ordinate of DHFR (PDB ID 4M6K) was downloaded from the protein data bank (www.rcsb.org). In the first step, bond orders were assigned and hydrogens were added by using pre-process option. All water molecules were deleted. The heteroatoms are ionized by epic at biological pH to consider the protein permeability and drug solubility and then the H-Bonds were optimized to reduce the steric clashes by histidine, aspartate, glutamate, and hydroxyl containing amino acids. Then complete protein structure was minimized by using OPLS 2005 force field. Ligand preparation is generally required because molecules lack 3D coordinates, ionization, stereochemistry and tautomer’s. Thus, before performing docking first the lowest energy state of ligand was generated using Ligprep tool of Schrodinger and then minimized with the help of the OPLS 2005 force field. For docking, the grids were generated by using the grid-based energy descriptor which had a default set of options with Van der Waals radius of 1.0. These grids were used to determine the interaction of prepared ligand with the receptor using the XP ligand docking in glide. Finally, Hydrogen Bonding, hydrophobic interactions and π-π stacking between enzyme and peptide were determined.

### Dihydrofolate Reductase Inhibition Assay

The dihydrofolate reductase inhibition assay was performed using DHFR assay kit (Sigma product code CS0340) according to the reported protocol. All the dilutions were made in assay buffer, pH 7.5. 10 mM stock solutions of dihydrofolic acid and NADPH in assay buffer were prepared. Stock solutions of the test peptides with different concentrations were prepared in DMSO, and an amount of 20 μL of each was taken to attain final concentration of 10^−9^, 10^−8^, 10^−7^, 10^−6^, 10^−5^, and 10^−4^ M in the respective wells of 96-well plate containing assay buffer. An amount of 0.1 unit of DHFR as supplied in the kit was diluted, and an amount of 20 μL of its 3 × 10^−3^ unit was used in each reaction. Each well of the 96-well plate was charged with 157.8 μL of assay buffer. Then 1.2 μL of NADPH solution was added to each well except 1 A and 1B, and 20 μL of test peptide (including methotrexate as positive control) was added to each well except 1 A–H. The reaction was started by the addition of 1 μL of dihydrofolic acid to each well except 1 C and 1D. 1 G and 1 H contained 20 μL of DMSO to check any inhibition of enzyme activity due to DMSO. The change in absorbance at 340 nm was monitored as a function of time. Percentage inhibition of enzymatic activity was calculated after nullifying the effects of NADPH, folate, and solvent. IC_50_ was calculated by plotting a graph between percentage inhibition and the corresponding concentration of the peptide.

### Cell Culture

A549, lung adenocarcinoma cell line, was purchased from ATCC and cultured in DMEM +10% FBS at 37 °C in 5% CO_2_, humidified environment.

### Cell viability (MTT assay)

Cell viability was measured using 3-(4,5-Dimethylthiazol- 2-yl)-2,5- Diphenyltetrazolium Bromide (MTT) assay. Briefly, a total of 5^103^ A549 cells were seeded, allowed to attach overnight and then treated with the specified concentration of peptide/peptide conjugated nanoparticles for 48 hours. Following that, 20 µL of 5 mg/ml of MTT reagent was added in a total volume of 200 µl and incubated for 2 hours. The culture media was removed and the formazan crystals so formed were dissolved in 200 µl of dimethyl sulfoxide (DMSO). Absorbance was measured at 570 nm (with 660 nm reference wavelength) using Tecan plate reader (Infinite M200 Pro). Each individual experiment was repeated at least thrice. Relative cell viability was expressed as a percentage relative to untreated control cells. IC_50_ values, which refers to the concentration required to inhibit 50% of cell proliferation, were calculated using non-linear regression analysis on graph pad prism.

### Live monitoring of cell viability (Propidium iodide staining)

A total of 5^103^ A549 cells were seeded in 96 well plates, allowed to adhere overnight and then treated with the specified concentration of peptide or nanoparticles along with 1 µg/ml of propidium iodide (PI). Cells were monitored for a period of 48 hours using Incucyte ZOOM live-cell imaging system (Essen Bioscience). Uptake of propidium iodide (PI) stain signifies loss of membrane integrity, and thereby allows monitoring of cell death. Percentage of cell confluence and no. of PI positive (dead) cells were plotted against elapsed time to monitor cell growth and cell death, respectively. Each experiment was done in triplicate.

## Electronic supplementary material

Supporting Information

Video S2

Video S1
