# Supplementary material for: Optimized peptide based inhibitors targeting the dihydrofolate reductase pathway in cancer
Source: Sci Rep. 2018 Feb 16;8:3190. doi: 10.1038/s41598-018-21435-5 (PMC5816602; doi:10.1038/s41598-018-21435-5)
Supplement: Supplementary file 1 — Supporting Information [file 41598_2018_21435_MOESM1_ESM.pdf]

Optimized peptide based inhibitors targeting the dihydrofolate reductase pathway in cancer

Amrinder Singh[1], Neha Deshpande[2], Nilkamal Pramanik[3], Siddharth Jhunjhunwala[3], Annapoorni Rangrajan[2]and Hanudatta S. Atreya[1*]

[1]*NMR Research Centre,*[2]*Department of Molecular Reproduction, Development and Genetics, and* [3]*Centre for Biosystems Science and Engineering, Indian Institute of Science, Bangalore-560012, India*

Characterization of peptides:

All the synthesized peptideswere characterized by mass and NMR spectral techniques. The mass spectra of the peptides are shown in Fig. S1.

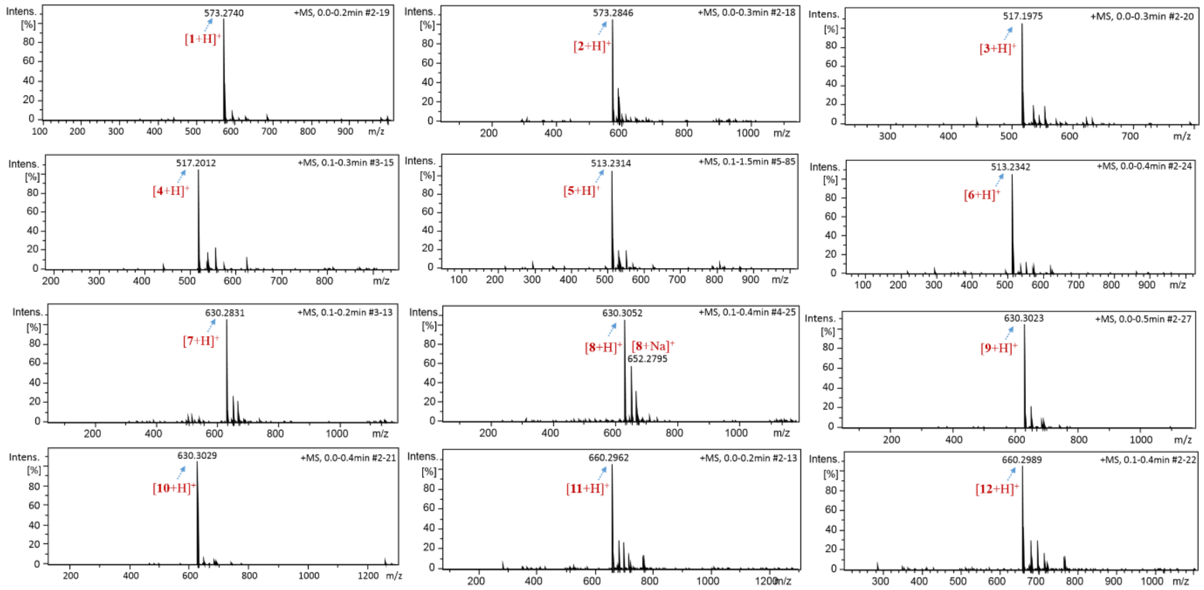

Fig. S1. Mass spectra of peptides.

The structure of all the peptides was confirmed by NMR spectroscopy. For instance, the ^{1}H and ^{1}H-^{1}H COSY spectrum of peptide **2** are shown in Fig. S2 and S3 respectively. The sequence specificity of different peptides was confirmed by using ROESY spectrum. First all resonances of amino acid residues in the peptide chain was assigned by employing ^{1}H-^{1}H TOCSY spectrum (Fig. S4a). The N-terminal amino group of peptide **2 (FMYL)**was not visible in the ^{1}H NMR spectrum possibly due to its broadening. The sequence of remaining amino acids (MYL) was ascertained by using combination of COSY and ROESY NMR spectrum (Fig. S4b).

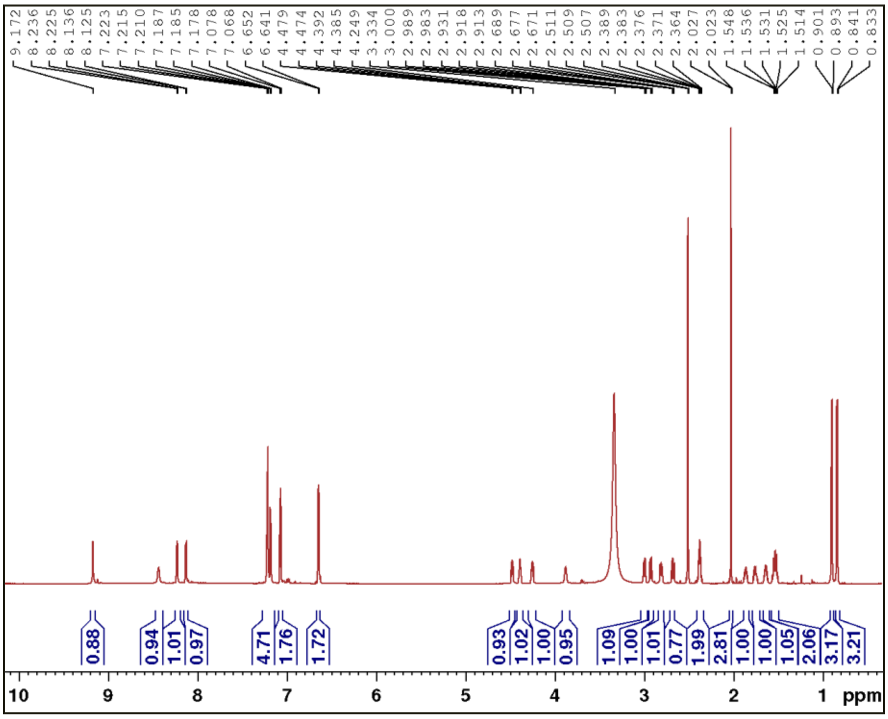

Fig. S2. ^{1}H NMR of peptide**2** in DMSO-d_6.

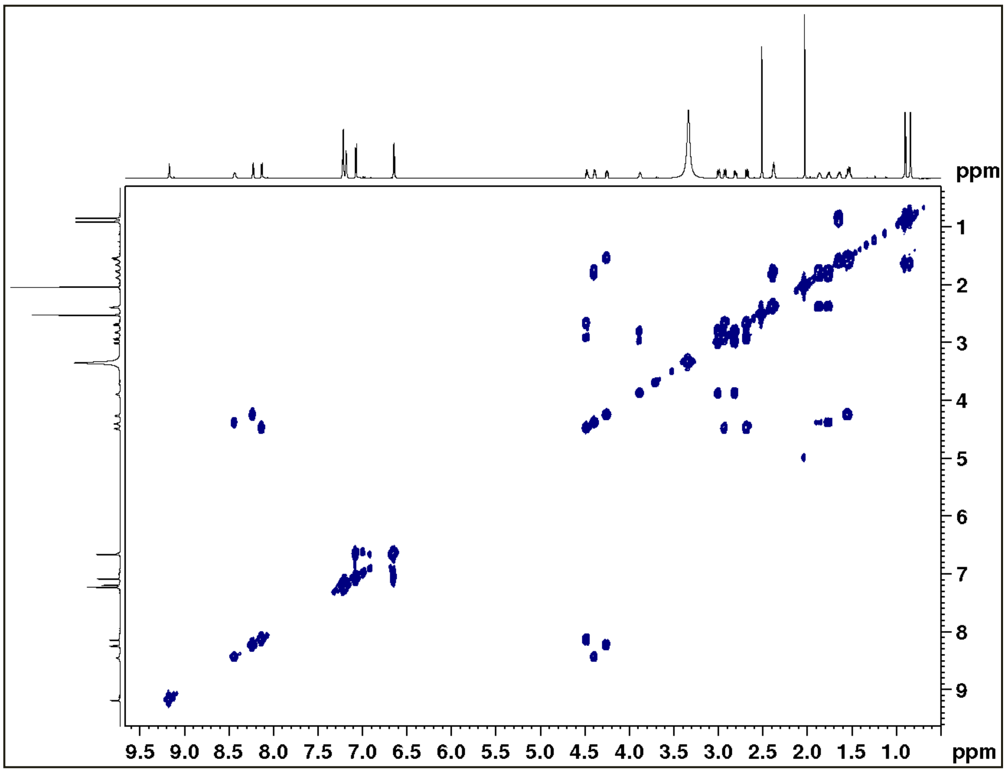

Fig. S3. ^{1}H-^{1}H COSY NMR spectrum of peptide**2**.

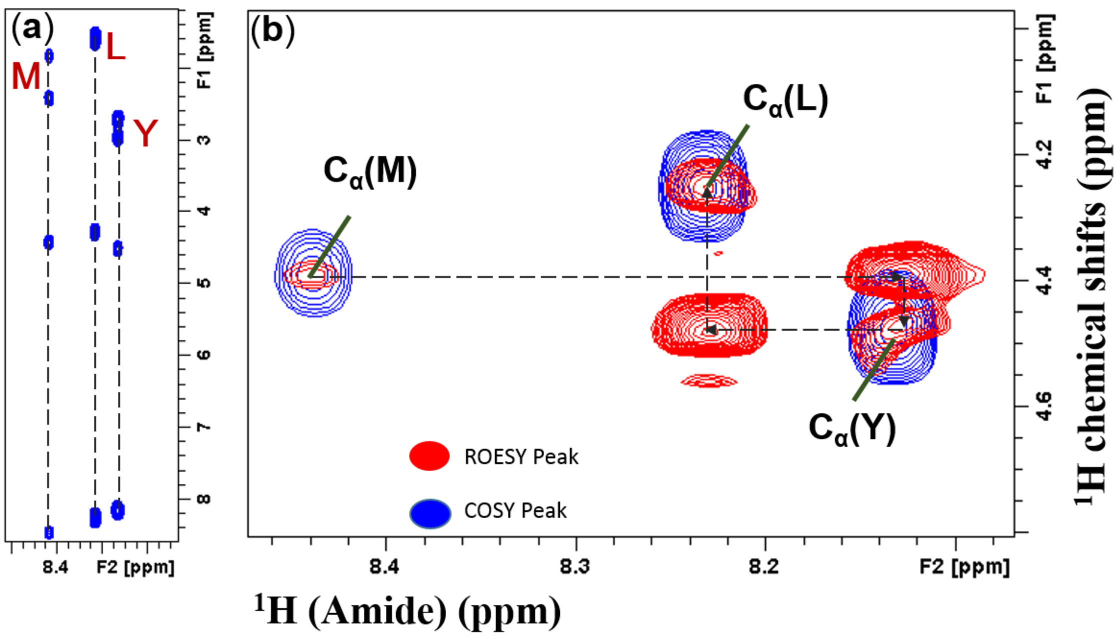

Fig. S4. (a) Part of ^{1}H-^{1}H TOCSY spectrum; (b) Overlay of COSY (blue) and ROESY (red) NMR spectra showing sequential connectivity.

Similarly, by following same approach the sequence specificity of peptide**11** (YSFML) was confirmed (Fig. S5).

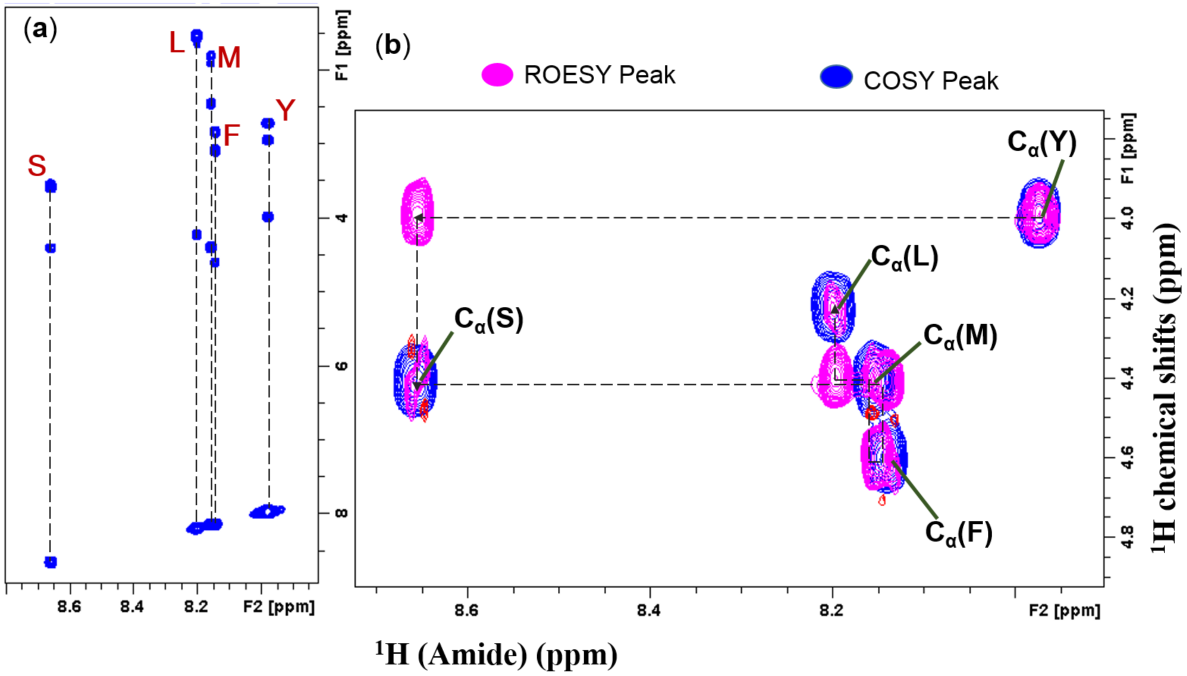

Fig. S5. (a) Part of ^{1}H-^{1}H TOCSY spectrum; (b) Overlay of COSY (blue) and ROESY (pink) NMR spectra showing sequential connectivity.

Materials:

Polystyrene nanoparticles with carboxy tail (diameter200nm, was purchased from Bangs laboratories. Inc, USA. 1-Ethyl-3-(3-dimethylaminopropyl) carbodiimide (EDAC), 2-(N-Morpholino) ethanesulfonic acid hemisodium salt (MES buffer) and N-Hydroxysuccinimide (NHS)were also purchased from SRL chemicals, India.

CHARACTERIZATION

Fourier transform infrared (FTIR) spectral analysis: The bond stretching frequency of each functional group present in the nanoparticles were recorded using a PerkinElmer FTIR spectrometer (model: L1860121, USA), scanning from 4000 cm^{-1} to 500 cm^{-1} for 42 consecutive scans at room temperature.

Size and zeta potential measurement: The hydrodynamic diameter and zeta potential values of the nanoparticles were measured using Zeta PALS, Zeta Potential Analyzer, Brookhaven Instruments Corporation at room temperature.

Atomic Force Microscopic (AFM) analysis: The surface morphology of the native carboxylated polystyrene nanoparticle, peptide conjugated polystyrene nanoparticles was investigated using an atomic force microscope (PARK SYSTEM, NX-10 AFM, XEI Software for imaging) in tapping mode at room temperature.

UV-Visible spectral analysis: The absorbance values of different concentrated peptide solutions, and the unreacted peptide extracts were recorded on UV-Visible spectrophotometer (Thermo Fisher Scientific, Nano Drop 1000) at a resolution of 1 nm.

Zeta potential measurement:

Zeta potential value of native polystyrene nanoparticles was found to be -30.14 mV. Modification of the particle surfaces with peptide results in a lowering of the surface potential to -8.51 mV. This result is expected, as peptide conjugation results in lowering of the number of free carboxyl groups that are the causative factor for high zeta potential values in the native nanoparticles.

Atomic Force Microscopy (AFM) analysis

Surface morphologies of native polystyrene and Pep-g-PS nanoparticles obtained by AFM analyses are shown in **Fig. S6**. Native polystyrene nanoparticles exhibited a population of homogeneous non-agglomerated particles of spherical shape with smooth surfaces. The size of discrete nanoparticles was found to be 180-200 nm (length/width and height). After

modification with peptide molecules via covalent conjugation, an increase in agglomeration was observed, and an associated increase in size to be 329 ± 81 nm (measured as the longest axis).

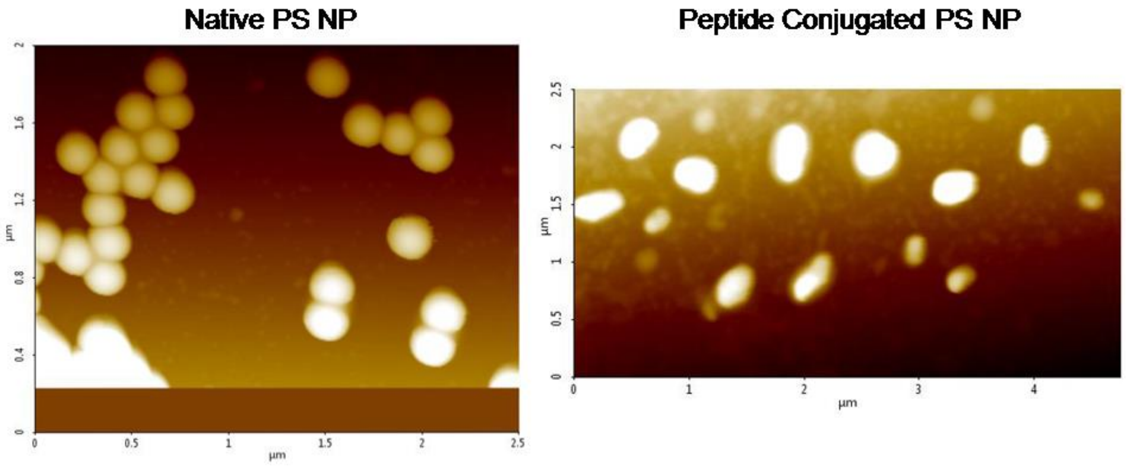

Fig. S6: AFM analyses of native polystyrene nanoparticles and peptide conjugated polystyrene nanoparticles

Attenuated total reflectance/Fourier transform infrared (ATR/FTIR) spectral analysis: ATR/FTIR spectrum of native and modified polystyrene nanoparticles was recorded to determine the changes due to peptide. Bond stretching vibration frequencies of different functional groups is shown in **Fig. S7.** In case of native polystyrene particles, the strong absorption peaks at 3308 cm^{-1}, 1722 cm^{-1}, and 1654 cm^{-1} assigned to the presence of stretching vibration bands of -OH group (asymmetric vibration), -COOH group and –C=C group present in the structure of polystyrene unit. Again, the peaks at 2927 cm^{-1} and 3056 cm^{-1}were attributed to the asymmetric stretching vibration of –C-H group in methylene and methine unit respectively. The additional bands at 745 cm^{-1} also confirmed the presence of aromatic units.
The solid state pathway synthesized peptide molecules also revealed a group of vibration bands at 3470 cm^{-1}, 1695 cm^{-1}, and 1635 cm^{-1}, indicating the presence of a strong asymmetric stretching band of $–NH_2$ group, -C=O group and $–CONH_2$ group in the peptide chain.
After conjugation of peptide molecules onto surface of polystyrene nanoparticles, the strong absorption band at 1722 cm^{-1} was found to be shifted at lower frequency values in the ranges of 1675-1640 cm^{-1} as compared to both peptide molecule and polystyrene molecule, leading to the formation of new amide linkage between the –COOH group of polystyrene unit and $–NH_2$ group of peptide unit. In addition, the peak around 3300 cm^{-1} was found to be increased to a

broad band peak at 3591 cm^{-1}, indicating the incorporation of additional amine (-NH_2group) into the modified polystyrene molecule.

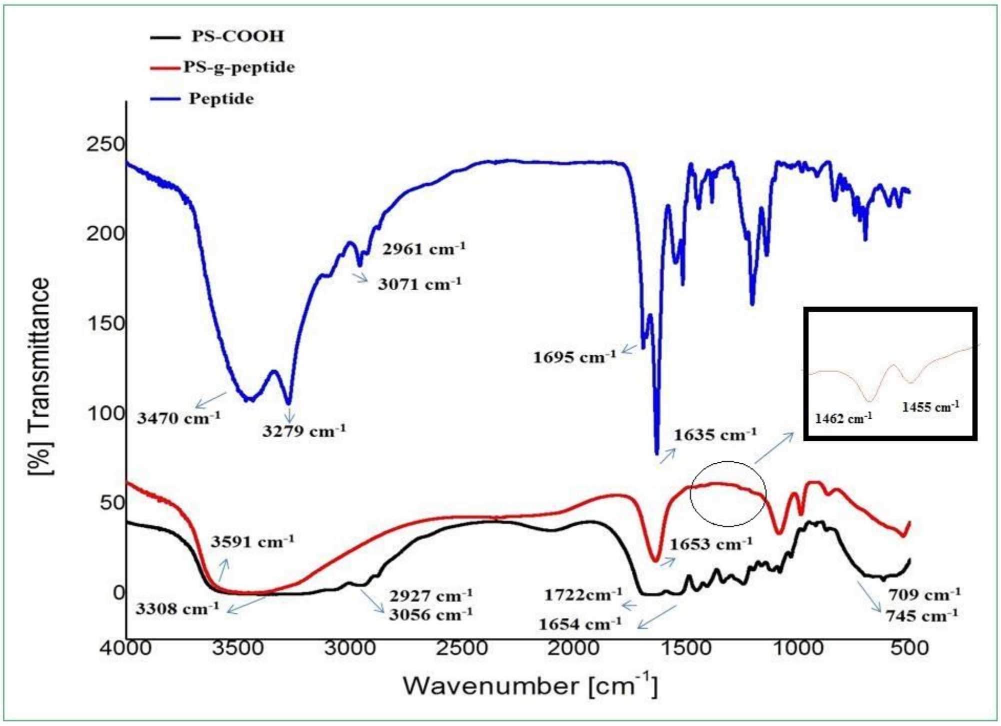

Fig. S7 ATR/FTIR analyses of synthesized peptide molecules, native polystyrene nanoparticles, and peptide conjugated polystyrene nanoparticles.

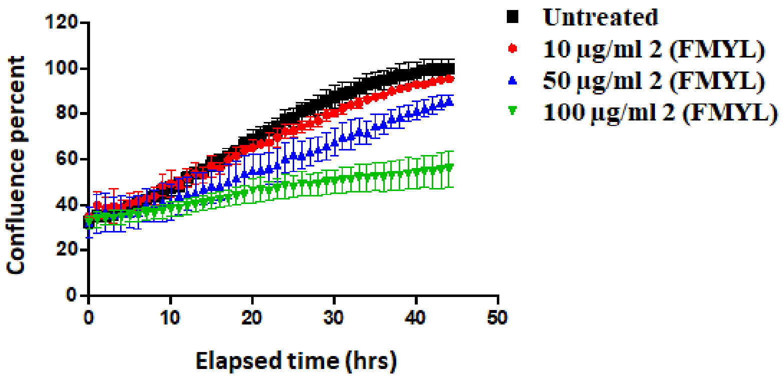

Fig. S8 Effect of increasing concentrations of peptide **2** on cell confluence percentage of A549 cells, as monitored using Incucyte ZOOM. Error bars represent mean±SEM; n=3.

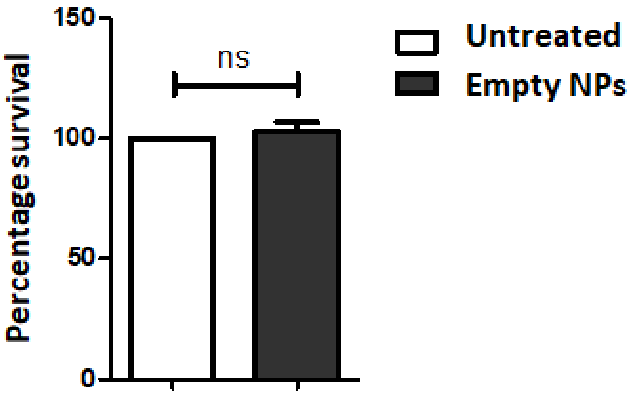

Fig. S9 Effect of empty polystyrene nanoparticles on cell viability of A549 cells following treatment for 48hrs, as assayed by MTT. Error bar represents mean±SEM, n=3.

Video S1 and S2: A549 cells, treated 1µg/ml of propidium iodide (PI) to monitor cell death, were either left untreated (Video S1) or treated with 100µg/ml of peptide **2** and imaged every hour for a period of 44hrs using Incucyte ZOOM imaging system
